# Optimal treatment scheduling of ionizing radiation and sunitinib improves the antitumor activity and allows dose reduction

**DOI:** 10.1002/cam4.441

**Published:** 2015-03-31

**Authors:** Esther A Kleibeuker, Matthijs A ten Hooven, Kitty C Castricum, Richard Honeywell, Arjan W Griffioen, Henk M Verheul, Ben J Slotman, Victor L Thijssen

**Affiliations:** 1 Department of Radiation Oncology, VU University Medical CenterAmsterdam, The Netherlands; 2 Department of Medical Oncology, VU University Medical CenterAmsterdam, The Netherlands

**Keywords:** Angiogenesis, combination therapy, preclinical tumor model, radiotherapy, sunitinib

## Abstract

The combination of radiotherapy with sunitinib is clinically hampered by rare but severe side effects and varying results with respect to clinical benefit. We studied different scheduling regimes and dose reduction in sunitinib and radiotherapy in preclinical tumor models to improve potential outcome of this combination treatment strategy. The chicken chorioallantoic membrane (CAM) was used as an angiogenesis in vivo model and as a xenograft model with human tumor cells (HT29 colorectal adenocarcinoma, OE19 esophageal adenocarcinoma). Treatment consisted of ionizing radiation (IR) and sunitinib as single therapy or in combination, using different dose-scheduling regimes. Sunitinib potentiated the inhibitory effect of IR (4 Gy) on angiogenesis. In addition, IR (4 Gy) and sunitinib (4 days of 32.5 mg/kg per day) inhibited tumor growth. Ionizing radiation induced tumor cell apoptosis and reduced proliferation, whereas sunitinib decreased tumor angiogenesis and reduced tumor cell proliferation. When IR was applied before sunitinib, this almost completely inhibited tumor growth, whereas concurrent IR was less effective and IR after sunitinib had no additional effect on tumor growth. Moreover, optimal scheduling allowed a 50% dose reduction in sunitinib while maintaining comparable antitumor effects. This study shows that the therapeutic efficacy of combination therapy improves when proper dose-scheduling is applied. More importantly, optimal treatment regimes permit dose reductions in the angiogenesis inhibitor, which will likely reduce the side effects of combination therapy in the clinical setting. Our study provides important leads to optimize combination treatment in the clinical setting.

## Introduction

With approximately 50% of all cancer patients receiving radiotherapy, this strategy is among the most commonly applied anticancer treatments worldwide 1,2. Apart from technical advances that continue to improve the accurate dose delivery to the malignant tissue, efforts are being made to develop drugs that increase the sensitivity of tumor cells to ionizing radiation (IR) 3–5. These radiosensitizers usually target cellular pathways that mediate radioresistance in tumor cells, for example, DNA repair mechanisms, cell cycle checkpoints, and cell survival signaling pathways 6,7. In recent years it has been suggested that drugs that inhibit tumor angiogenesis, that is, the growth of tumor blood vessels, can also potentiate the antitumor effect of IR. Indeed, this combination has demonstrated promising results in animal tumor models in vivo 8–10. However, how both treatment modalities should be scheduled to obtain the maximum antivascular and antitumor effect is still poorly understood 11.

Sunitinib (Sutent, SU11248) is a receptor tyrosine kinase (RTK) inhibitor that targets multiple receptors involved in angiogenesis, including vascular endothelial growth factor receptor (VEGFR)-1, -2, and -3 and platelet-derived growth factor receptor (PDGFR) 12. Treatment with sunitinib is currently approved by the FDA for different cancer types, including metastatic renal cell carcinoma and certain gastro-intestinal stromal tumors. Several preclinical in vivo studies that combined sunitinib with IR have demonstrated promising antitumor effects 13–15. In addition, a number of phase I/II clinical trials have shown that this combination is a generally well-tolerated combination therapy with promising tumor response rates 16–19. However, there is a major concern about rare but severe side-effects, such as hemorrhages or gastro-intestinal perforations 18,20. While some preclinical studies have demonstrated that precise scheduling of the two treatment modalities is essential for the antitumor effect, it is still poorly understood if optimal scheduling also permits dose reductions in either treatment modality 13,15. This is an important issue to address, as dose reductions could lead to lower toxicity. This is supported by clinical trials in which decreased sunitinib doses resulted in lower toxicity rates 16,19 and by case reports that observed no change in response upon dose reduction 21,22. This warrants more preclinical research to resolve the optimal dose-scheduling of radiotherapy and sunitinib.

In this study, we transplanted human tumors on the chorioallantoic membrane (CAM) of the chicken embryo to study the effects of IR and sunitinib on angiogenesis and tumor growth in vivo. In addition, we evaluated whether proper scheduling would allow dose reductions in either sunitinib or IR. Our data show that optimization of dose-scheduling enhances the effects of IR and sunitinib on angiogenesis and tumor growth. More importantly, optimal dose-scheduling allows dose reduction in sunitinib by at least 50% while maintaining the same antitumor effect. In addition, by adding sunitinib to IR, the dose of IR can be reduced while maintaining the same antitumor effect as IR alone. Altogether, our results demonstrate that the combination of IR and angiogenesis inhibition can be improved by optimizing the dose and scheduling of both treatment modalities.

## Materials and Methods

### Cell culture

Cancer cell line HT29 (colon carcinoma) was cultured in DMEM (Lonza, Breda, The Netherlands) and OE19 (esophageal adenocarcinoma) in RPMI (Lonza), both supplemented with 10% fetal calf serum (FCS) and 1% Penicillin-Streptomycin (Lonza) and for OE19 (kindly provided by Dr. H van Laarhoven, Amsterdam Medical Centre, The Netherlands) with 1% l-glutamine (Invitrogen, Leusden, The Netherlands) in a 37°C humified incubator, with 5% CO_2_. Both cell lines were authenticated before start of the experiments and with were repeatedly found negative for mycoplasm infection as checked by PCR.

### Proliferation assay

Six hours after plating 2 × 10^3^ HT29 cells/well in a 96-well plate, 4 Gy IR and sunitinib was applied. Nonirradiated cells served as a control and for each condition a minimum of 4 wells were plated. On indicated time points, the amount of viable cells was determined, adding 100 *μ*L CellTiter-Glo (Promega, Leiden, The Netherlands) reagent to the cells. After the suspension was incubated for 20 min at room temperature (RT), luminescence was measured. Experiments were performed in duplicate, with a minimal of four replicates in each experiment.

### Analysis of cell cycle and apoptosis

In a 6-well plate, 2 × 10^5^ HT29 cells/well were plated and 6 h later 4 Gy IR was applied. Nonirradiated cells served as a control. On indicated time points, cells were collected with trypsin EDTA (Lonza), resuspended in 70% ethanol and stored in −20°C for at least 2 h for fixation. After spinning cells at 400 g for 5 min, ethanol was discarded and cells were then incubated in DNA extraction buffer (90 parts 0.05 mol/L Na_2_HPO_4_.2H_2_O, 10 parts 0.025 mol/L citric acid, 1 part 10% Triton X-100) for 20 min at 37°C. Propidium iodide was then added (20 *μ*g/mL) and cell cycle distribution was measured using flow cytometry 23. Experiments were performed in duplicate.

### Chorioallantoic membrane assay

Fertilized white leghorn chicken eggs were incubated at 38°C in a fan-assisted humidified egg incubator. From embryonic development day (EDD) 0 to EDD3 the eggs were placed horizontally, rotating 90° each hour. On EDD3 eggs were put in an upright position and with fine tweezers a hole was made in the narrow end of the shell. On EDD6 a treatment window of ±1 cm^2^ was created which was sealed with adhesive tape.

For drug treatment experiments without tumor xenografts, a nonlatex dental elastic ring (Ø 9.5 mm) was carefully applied onto the CAM on EDD6. Antiangiogenesis treatment consisted of daily application of the indicated concentrations in 50 *μ*L within the ring. Sunitinib (20 mmol/L in DMSO, Pfizer, Cappele a/d IJssel, The Netherlands) was diluted in 0.9% saline, as a control 0.9% saline with the required concentration of DMSO was used.

At the end of each experiment, the chicken embryos were first killed by hypothermia (30 min at 4°C). After injection of ±1 mL contrast agent (zinc oxide in pure vegetable oil) under the CAM, pictures were taken with a Leica DFC425 camera mounted on a Leica M125 microscope. Multiple vascular parameters, including vessel length, vessel area, number of branching points, and number of endpoints were quantified in each picture using CAM analysis software (HetCAM, DCILabs, Keerbergen, Belgium). All experiments were performed on a minimal number of eight eggs in at least two independent experiments.

### CAM tumor grafts

For tumor growth experiments on the CAM, 5 × 10^6^ HT29 cells or 7.5 × 10^6^ OE19 cells were resuspended in 50 *μ*L cold growth factor reduced Matrigel (Becton Dickinson, Breda, The Netherlands) and kept on ice until grafting. After slightly lacerating a small area of the CAM with a soft tissue, the cell-matrigel suspension was applied to the CAM on EDD6. The eggs were incubated under standard conditions and any subsequent treatment of the tumor started on EDD10. The sunitinib in saline was applied topically on the CAM close to but not directly onto the tumor at the indicated dose. Tumor size was measured each day and the volume was calculated as follows: *(length)*^2^*width*0.5, with length and width in mm. At the end of each experiment the tumors were harvested and weighed. Tumors were stored in ZincFix (0.5 g calcium acetate, 5.0 g zinc acetate, 5.0 g Zinc Chloride in 1 L of 0.1 mol/L Tris Buffer, pH 7.4). Subsequently, the tissues were paraffin embedded according to standard procedures. Each experiment was performed with a minimum of nine tumors per group.

### Ionizing radiation

The cells and eggs were irradiated on the indicated day at RT by *γ*-radiation using a ^60^Co source (Gamma Cell 200; Atomic Energy of Canada, Chalk River, Canada). Following IR, the eggs were routinely checked on a daily basis.

### Immunohistochemistry

Immunohistochemical (IHC) stainings were performed on 4-*μ*m-thick paraffin sections of CAM tumors. Following deparaffinization in xylene and rehydration through a graded series of alcohol, endogenous peroxidase activity was blocked by 20 min incubation in 0.3% H_2_O_2_/PBS. Next, antigen retrieval was performed in sodium citrate solution (pH 6.0) using a pressure cooker. After a blocking step of 30 min in 5% BSA/PBS at RT, the samples were incubated for 1 h at RT with the primary antibody diluted in 0.5% BSA/PBS. The following primary antibodies were used: CD31 (SZ31, Dianova, Huissen, The Netherlands), cleaved caspase-3 (5A1E, Cell Signal Technology, Leiden, The Netherlands), and Ki-67 (M7240; Dako, Heverlee, Belgium). Control slides were incubated with 0.5% BSA/PBS. Next, the slides were incubated for 30 min at RT with the appropriate secondary biotinylated antibody, followed by incubation with strep-ABC-HRP for 30 min at RT (1 *μ*L avadin and 1 *μ*L biotin in 500 *μ*L PBS). Finally, staining was visualized with 3,3-diamino-benzidine-tetra hydrochloride (DAB, 0.3 mg/mL) in 1 mL PBS with 0.3% H_2_O_2_. All slides were counterstained with hematoxylin and mounted in Entellan (Merck, Amsterdam, The Netherlands) for microscopy. A minimum of 4 pictures of each slide were taken at 100× magnification and quantification of positive DAB staining was performed using Image J with color deconvolution 24.

### Measuring intratumoral and circulating sunitinib concentrations

Sunitinib concentration in tumors dissected from the CAM and in peripheral blood, withdrawn from a CAM vein, was determined by liquid chromatography tandem mass spectrometry (LCMS) as described previously 25.

### Statistical analysis

All data are shown as mean ± standard error of mean (SEM), unless indicated otherwise. For statistical analysis of the CAM angiogenesis experiments the Mann–Whitney rank sum test was used. The two tailed Student’s *t*-test for was applied for IHC quantification and the two-way analysis of variance for tumor growth experiments with the post hoc multiple comparison Bonferroni test. *P*-values <0.05 were considered statistically significant.

## Results

### Ionizing radiation has a fast but transient effect on angiogenesis

**Figure 1:**
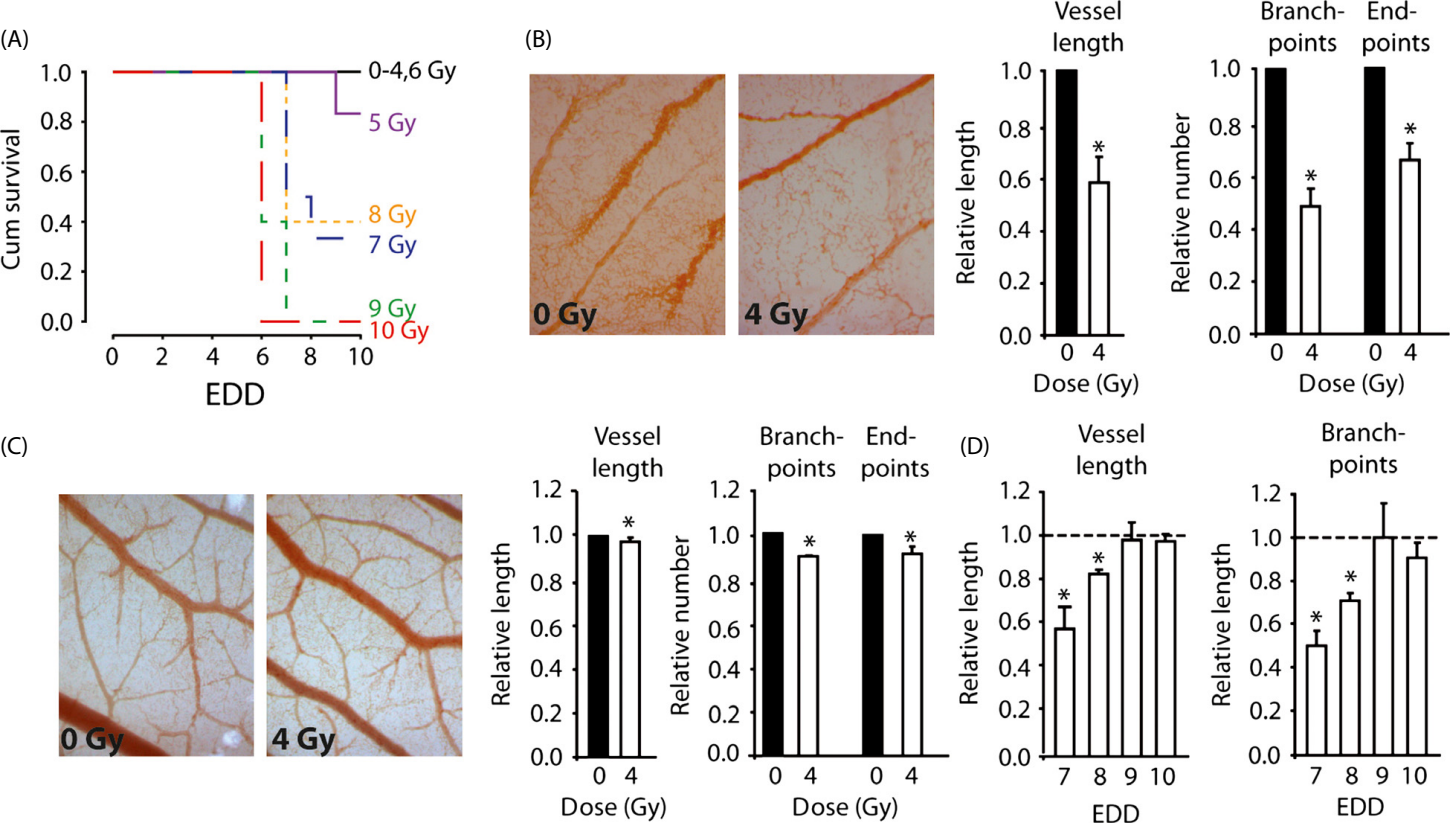
Effect of ionizing radiation on the vasculature in the chorioallantoic membrane (CAM). (A) Survival curves showing dose-dependent embyronic lethality after single-dose IR. The maximal tolerated dose was 4 Gy. (B) Effect of 4 Gy IR on the microvasculature in vivo on EDD7. Images (100× magnification) were obtained 24 h following IR and vascular parameter were quantified using HetCAM software. *P < 0.05 versus 0 Gy. (C) Effect of 4 Gy IR on the microvasculature in vivo on EDD12. Images (100× magnification) were obtained 24 h following IR and vascular parameter were quantified using HetCAM software. *P < 0.05 versus 0 Gy. (D) Time course experiment showing rapid recovery of the microvascular bed following single-dose IR (4 Gy).

We set out to study the effects of scheduling the combination of IR with angiogenesis inhibitors in vivo. For this, we used the chicken CAM assay, which is commonly used in angiogenesis research to evaluate the efficacy of angioregulatory drugs 26–29. The CAM is a highly vascularized membrane that facilitates gas exchange between the growing chicken embryo and the environment. It develops unidirectional from embryonic day of development (EDD) 3 to EDD10 (Fig. S1A). Subsequently, endothelial cell proliferation decreases and vessel maturation occurs 30. This was confirmed by analysis of different vascular parameters within the established macro- and microvascular bed throughout CAM development (Figs. S1B and C). To determine whether the CAM could be used to study the effects of IR, we first performed a dose escalation study (0-10 Gy) at EDD6. This resulted in a dose-dependent increase in embryonic lethality with 4 Gy as the maximal tolerable dose (Fig.1A). Analysis of the vascular parameters 24 h after 4 Gy showed an almost 50% reduction in the vascular parameters, including vessel length, number of branchpoints, and number of endpoints in the capillary bed (Fig.1B). No changes were observed in the macrovascular bed, that is, the established vessels (data not shown). Furthermore, when 4 Gy was applied at EDD12 there was only a 7–10% reduction in the vascular parameters (Fig.1C). All this confirms previous observations that growing and immature blood vessels are more sensitive to IR as compared to mature blood vessels 31. Finally, we determined whether the effects of irradiation sustained. Time series experiments showed that all vascular parameters normalize to the level of the nonirradiated CAM within 3 days following 4 Gy (Fig.1D). Altogether, these data identify the CAM as a suitable model to study the fast and transient effects of IR on growing vessels.

### Sunitinib potentiates the effect of IR on angiogenesis

**Figure 2:**
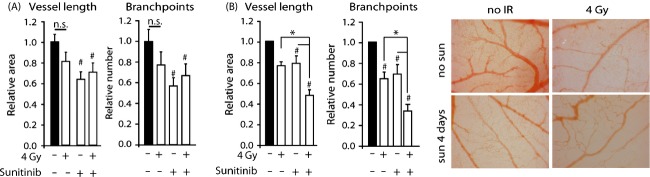
Effect of combination therapy on the microvasculature in the CAM. (A) Effect of IR with or without 4 days of sunitinib on vascular parameters. Sunitinib was applied from EDD6-9. Single-dose IR was applied on EDD 6, 4 h before the first sunitinib or control treatment. Images of the CAM were acquired and analyzed on EDD10. ^#^*P* < 0.05 vs untreated. (B) Similar as in (A) but now IR applied on EDD9, 4 h after last sunitinib treatment ^#^*P* < 0.05 vs untreated. *P < 0.05 versus indicated. (C) Representative CAM images on EDD10 showing the effects of 4 Gy IR on EDD9 or sunitinib on the vascular bed.

Next, we set out to combine IR with angiostatic therapy. For the latter, the FDA-approved drug sunitinib was used, a tyrosine kinase inhibitor mainly targeting VEGF- and PDGF receptors. First, the effect of sunitinib as a monotherapy was evaluated. Dose–response experiments identified 5.3 *μ*g/mL sunitinib (50 uL, daily application) to effectively inhibit vascular development in the CAM (Fig. S2A). Similar to IR, sunitinib only affected the microvasculature and the effects normalized to the level of the nontreated CAM within 4 days after treatment (Fig. S2B and C). Of note, 2 days of sunitinib treatment (EDD6 + 7) resulted in similar but smaller effects (Fig. S2D). Next, we tested the effect of IR preceding sunitinib, with 4 Gy administered on EDD6 and sunitinib from EDD6-9. Measuring vascular parameters on EDD10 did not reveal any effects of IR alone while the combination was as effective as sunitinib alone (Fig.2A). To further address the importance of scheduling, next we applied IR (EDD 9) after sunitinib treatment for 4 days (EDD6-9). This schedule reduced the microvasculature more than either treatment alone (Fig.2B). These data illustrate the importance of scheduling both treatment modalities in order to obtain maximal efficacy.

### Ionizing radiation reduces the tumor growth of human tumors cells on the CAM and transiently affects the tumor vasculature

**Figure 3:**
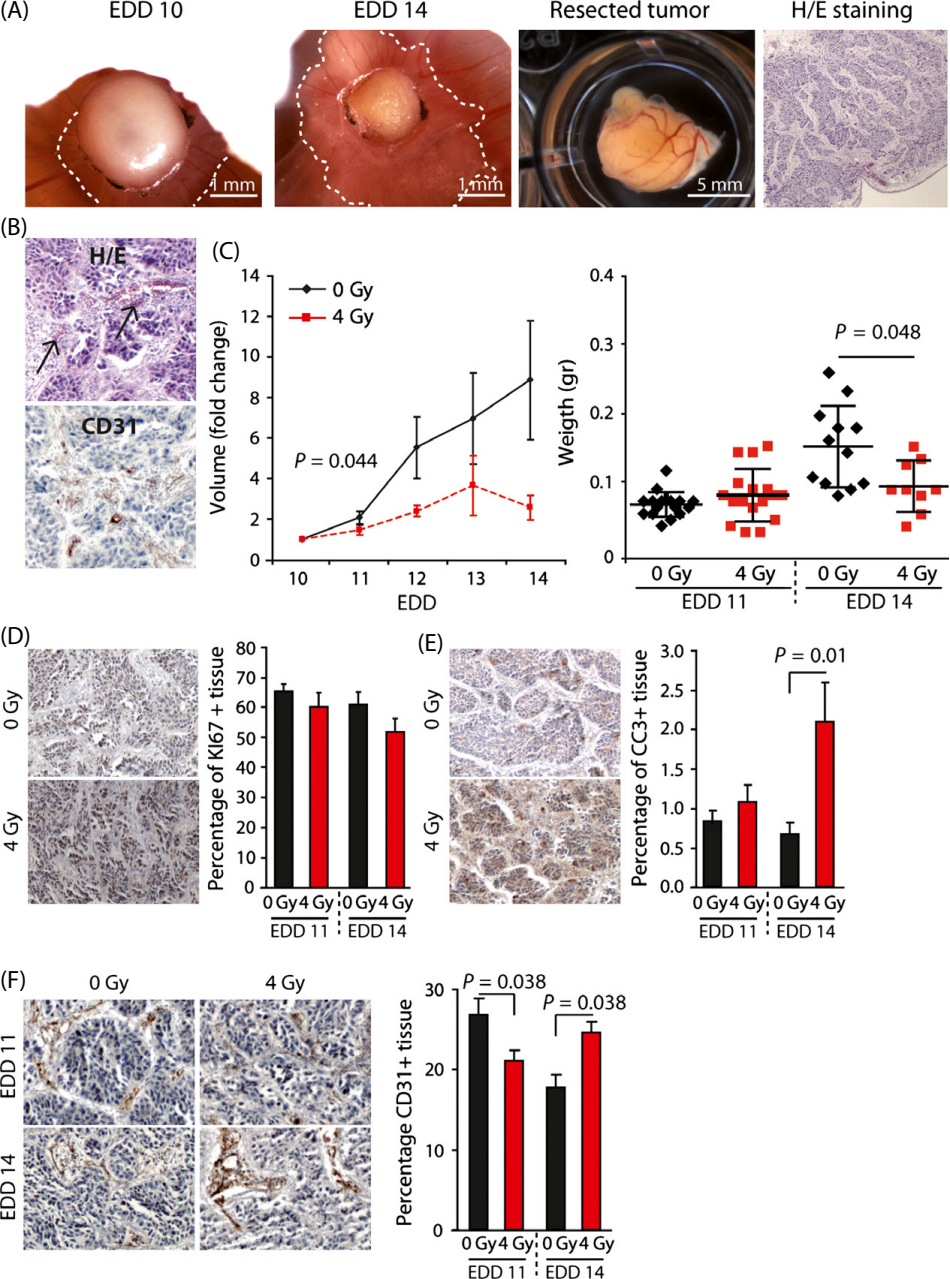
Effect of ionizing radiation on HT29 tumor growth in vivo. (A) Representative images of HT29 tumor grafts on the CAM. The two panels on the left show the same tumor on EDD10 and EDD14. The ‘cap’ on top of the CAM consists of Matrigel and cell debris. The viable tumor is growing just beneath the CAM (dotted lines). The two panels on the right show a resected tumor on EDD17 and H/E staining on a resected tumor with clear tumor cell nests surrounded by stromal tissue. (B) H/E staining showing the presence of red blood cells (arrows) in the tumor tissue (upper panel). IHC showing CD31 +  (brown) vessels within the tumor tissue (lower panel). (C) Tumor growth curves showing growth inhibition following single-dose IR (4 Gy) on EDD10. The bar graph shows the average weight of tumors resected either on EDD11 or EDD14 (average + SD, n ≥ 9). (D) IHC stainings (left) for Ki67 (proliferation marker). The bar graph shows the quantification of Ki67 +  cells in control and irradiated tumors that were resected either on EDD11 or EDD14. Quantification was performed using ImageJ with color deconvolution. (E) Similar as in (D), but now staining was performed for cleaved caspase 3 (CC3, apoptosis marker). (F), Similar as in (D), but now staining was performed for CD31 (endothelial cell marker).

To investigate the effects of dose-scheduling on tumor angiogenesis and tumor growth, we next grafted HT29 human colon carcinoma cells on the CAM at EDD6. On EDD10 tumors had reached a volume of ±18 mm^3^. The tumors predominantly grew just below the CAM surface and hematoxylin/eosin (H/E) staining of resected tumors revealed an organized structure of clear tumor cells nests surrounded by stromal compartments (Fig.3A). The tumors also showed the presence of red blood cells, indicative of tumor vascularization which was confirmed by CD31 staining (Fig.3B). Furthermore, the tumors showed a clear response to IR as reflected by a decrease in tumor volume and weight 4 days after 4 Gy (Fig.3C). A comparable response was observed in tumors of a human esophageal adenocarcinoma cell line OE19 (Fig. S3). Ionizing radiation appeared to induced a decrease in proliferating cells in the HT29 tumors (Fig.3D). While did this not reach significance when scoring the number of Ki-67^+^ cells by IHC, in vitro data confirmed significantly lower proliferation rates 4 days after IR (Fig. S4A). Furthermore, in vitro analyses also showed a G2/M and S phase arrest, respectively, after 4 Gy (Fig. S4B). This suggests an overestimation of proliferating cells with Ki-67 IHC in the tumors as described previously 32,33. The apoptotic fraction was not enhanced 24 h after 4 Gy IR, but an increase was observed 4 days after IR (EDD 14) (Fig.3E). This was in line with in vitro analyses (Fig. S4C), Together, these mechanisms likely underlie the decrease in tumor volume by IR.

Of note, we detected a decrease in endothelial cells 24 h after 4 Gy (EDD11). Interestingly, the reduction in the vessel density in nonirradiated tumors, which is caused by vessel growth lagging behind tumor expansion, was reversed in IR tumors (Fig.3F). Thus, while IR impairs tumor growth, the initial inhibition of vessel growth rapidly recovers, similar as observed in our previous experiments.

### Sunitinib reduces the tumor growth on the CAM by reducing proliferation and inhibiting angiogenesis

**Figure 4:**
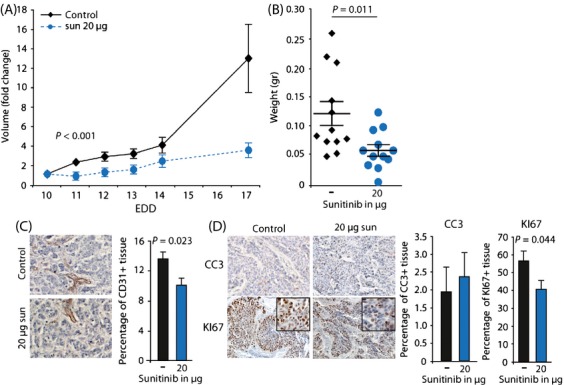
Effect of sunitinib on HT29 tumor growth in vivo. (A) Growth curves of HT29 tumors grafted on the CAM on EDD6. Treatment with sunitinib (20 ug/day in 50uL) was applied from EDD10-13. (B) Weight of tumors resected on EDD17 (average + SEM, n ≥ 9). (C) IHC stainings for CD31 (endothelial cell marker). The bar graph shows the quantification of CD31 +  cells in control and sunitinib treated tumors that were resected on EDD17. Quantification was performed using ImageJ with color deconvolution. (D) Similar as in (C) but staining was performed for cleaved caspase 3 (CC3, apoptosis marker) or Ki67 (proliferation marker).

To determine the effect of monotherapy with sunitinib on tumor growth, first a dose-safety study was performed. Determining the intratumor sunitinib concentration with LCMS demonstrated that topical administration of sunitinib on the CAM close to the tumor resulted in an almost four times higher sunitinib concentration as compared to direct intravenous administration (82 nmol vs 21.8 nmol) 24 h after 1 dose of 160 *μ*g sunitinib. Thus, while sunitinib was applied topically on the CAM vasculature, it acted systemically. The systemical effect was also confirmed by observed toxicities in the chicken embryos (black necrotic claws, recognized as the hand-foot syndrome) 48 h after sunitinib application. Due to the toxicities, the dose had to be reduced to 50% divided over 4 days (EDD10-13), that is, 20 *μ*g sunitinib per day, corresponding to 32.5 mg/kg/day, as the maximum tolerable dose. This treatment schedule significantly reduced tumor growth and weight in HT29 tumors (Figs.4A and B) as well as in OE19 tumors (Fig. S5). In line with the described angiostatic activity, sunitinib treatment significantly reduced the percentage of CD31^+^ cells (Fig.4C). In contrast to IR, sunitinib treatment did not increase the apoptotic fraction but decreased the percentage of proliferating tumor cells (Fig.4D), which was confirmed in vitro (Fig. S4D). These data not only confirm that sunitinib monotherapy inhibits tumor growth via angiogenesis inhibition and tumor cell growth but also indicate that sunitinib treatment might be complementary to IR.

### Ionizing radiation given before sunitinib effectively inhibits tumor growth

**Figure 5:**
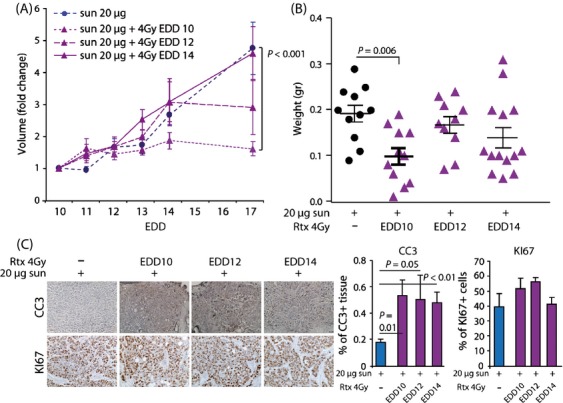
Effect of scheduling combination therapy on HT29 tumor growth in vivo. (A) Growth curves of HT29 tumors grafted on the CAM on EDD6. Treatment with sunitinib (20 ug/day in 50uL) was applied from EDD10-13. Ionizing radiation (4 Gy) was applied on either EDD10, EDD12 or EDD14. (B) Weight of control and treated tumors resected on EDD17 (average + SEM, n ≥ 10). (C) IHC stainings for CC3 (apoptosis marker) and KI67 (proliferation marker). The bar graph shows the quantification in tumors resected on EDD17. Quantification was performed using ImageJ with color deconvolution.

To study the possible complementarity of both treatment modalities, we next combined 4 Gy IR with 20 *μ*g sunitinib using three different treatment schedules. The tumors in the control group received sunitinib from EDD10-13. In the additional three groups the tumors also received 4 Gy IR, either on EDD10 (neo-adjuvant), EDD12 (concurrent), or EDD14 (adjuvant). The combination of sunitinib with preadjuvant IR resulted in an almost complete inhibition of tumor growth and significant reduction in tumor weight (Fig.5A and B). The concurrent IR inhibited tumor growth to a lesser extent, whereas adjuvant IR did not further affect tumor growth or weight (Fig.5A and B). Again, we observed an induction of apoptosis after 4 Gy IR, whereas Ki-67 IHC did not demonstrate a difference in proliferating fraction (Fig.5C). These experiments illustrate that proper scheduling of combination therapy with sunitinib and IR can almost completely block tumor growth.

### Optimal scheduling allows dose reduction in sunitinib without affecting therapeutic efficacy

**Figure 6:**
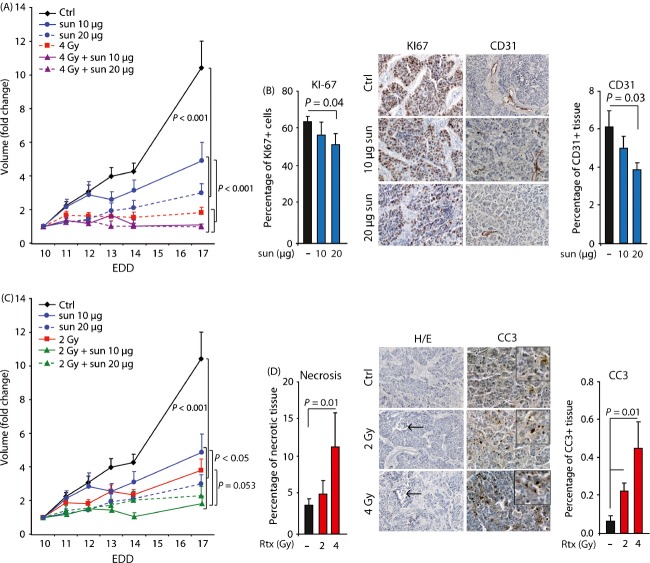
Effect of dose reduction in either IR or sunitinib on HT29 tumor growth in vivo. (A) Growth curves of HT29 tumors on CAM subjected to different treatment regimes. Treatment with sunitinib (10 or 20 ug/day in 50uL) was applied from EDD10-13. Ionizing radiation (4 Gy) was applied on EDD10. (B) Quantification of proliferation (left, Ki67 staining) and vessel density (right, CD31 staining) following either 10 ug/day or 20 ug/day sunitinib. (C) Growth curves of HT29 tumors on CAM subjected to different treatment regimes. Treatment with sunitinib (10 or 20 ug/day in 50uL) was applied from EDD10-13, IR (2 Gy) was applied on EDD10. (D) Quantification of necrosis (left, H/E staining) and apoptosis (right, cleaved caspase 3 staining) following either 2 Gy or 4 Gy IR. Arrows indicate area of necrosis. n ≥ 9 for growth curves, n ≥ 5 for IHC analysis.

Finally, we determined whether optimal scheduling would allow dose reduction in sunitinib. We reduced the dose of sunitinib with 50% to 10 *μ*g/day for 4 days. As expected, monotherapy with 10 *μ*g sunitinib had less effect on tumor growth than 20 *μ*g sunitinib (Fig.6A). This was accompanied by less inhibition of the CD31^+^ fraction and tumor cell proliferation (Fig.6B). However, when 10 *μ*g sunitinib was combined with 4 Gy IR, this also resulted in a complete inhibition of the tumor growth, comparable to 20 *μ*g sunitinib with 4 Gy IR (Fig.6A). While the combination treatment was significantly more effective than 10 *μ*g sunitinib alone, no statistically significant difference to 4 Gy alone was observed. This is most likely due to the considerable effect of 4 Gy alone and the limited follow up time due to hatching of the eggs. Therefore, we also reduced the dose of IR with 50% to 2 Gy. Though the timing of 2 Gy IR (EDD10, 12 or 14) had no significant influence on reducing the tumor growth (Fig. S6A), 2 Gy induced a significant inhibitory effect on tumor growth, albeit less as compared to 4 Gy (Fig.6C and Fig. S6B). This was also reflected by the percentage of necrotic tissue and apoptotic cells in these tumors (Fig.6D). When 2 Gy IR in the preadjuvant schedule was combined with 20 *μ*g sunitinib an additional reduction in tumor growth was achieved (Fig.6C). Next, we reduced the dose of sunitinib by 50% to 10 *μ*g/day for 4 days. The monotherapy of 10 *μ*g sunitinib was less effective than 2 Gy IR, while the combination was similarly effective as 20 *μ*g sunitinib + 2 Gy IR. In addition, 10 *μ*g sunitinib + 2 Gy IR was significantly more effective then sunitinib alone and reached borderline significance with 2 Gy IR (Fig.6C). Of note, both combinations had comparable effects as 4 Gy IR alone (Fig. S6C). We also applied the combination of this suboptimal treatment on grafted OE19 cells. While monotherapy with either 10 *μ*g sunitinib (EDD10-13) or 2 Gy IR (EDD10) resulted in a tumor growth reduction in already approximately 50% compared to the nontreated tumors, combining the two treatment modalities resulted in a tumor growth reduction of nearly 80%. This was also reflected in tumor weight (Fig. S7).

These data demonstrate that optimization of dose-scheduling allows halving the dosage of sunitinib without loss of therapeutic efficacy.

## Discussion

Despite encouraging preclinical studies that combine radiotherapy with angiostatic drugs, varying results and increased side effects in the clinical setting demonstrate that more research is warranted to optimize this combination treatment. We therefore studied the effects of dose-scheduling of IR and the clinically available angiostatic drug sunitinib on both angiogenesis and tumor growth. Our observations show the importance of proper scheduling of both treatment modalities in order to obtain maximum treatment effects. Moreover, our data demonstrate that optimal scheduling allows dose reductions in sunitinib while maintaining the same antitumor effect. The latter is clinically relevant as it could reduce side-effects in patients without affecting treatment efficacy. This is in line with two case reports in which sunitinib dose reductions did not affect the clinical benefit of the treatment 21,22.

In this study, we demonstrate that the CAM assay is a feasible method to study the effect of combination therapy on both angiogenesis and tumor growth. Both IR and sunitinib had a significant inhibitory effect on angiogenesis. In addition, both treatments significantly reduced tumor growth. As expected, IR had a dose-dependent effect on tumor cell apoptosis and proliferation while sunitinib reduced the microvessel density (MVD) in the tumors as well as the tumor cell proliferation. This is in line with previous observations in different mouse tumor models, where it was shown that sunitinib not only has a direct effect on the tumor vasculature, but also on the tumor cells 34–36. All these observations identify the CAM tumor model as a representative model to study (tumor) angiogenesis and tumor growth in vivo. A potential limitation of the CAM tumor model is the relatively short time frame during which therapy can be applied. Nevertheless, our current data show that already within this time frame comparable observations can be made as in, for example, mouse tumor models. Moreover, as the model is reliable and affordable it provides a good alternative for rapid drug screening or monitoring the efficacy of (combination) therapy. The latter is illustrated in this study.

While the effect of a limited number of drugs in combination with IR has been studied on the CAM 37,38, we now demonstrate that the efficacy of combining IR with sunitinib is dependent on the schedule that is used. In agreement with others, we show that IR alone reduces angiogenesis and that this recovers within 96 h 37–39. Interestingly, our data suggest that the effect of IR on the vasculature is also transient when IR is followed by sunitinib treatment, as this combination treatment schedule was as effective as sunitinib alone. On the other hand, we observed that IR applied after sunitinib treatment was more effective than either treatment alone. While this already shows the importance of proper scheduling, the opposite effect of scheduling was observed in the tumor model. Here, IR given after sunitinib had no additional effect on tumor growth compared to sunitinib alone, whereas IR given before sunitinib stopped tumor growth completely. This not only further exemplifies the importance of scheduling, it also shows the importance of the environmental context, for example, normoxic CAM versus hypoxic tumor tissue, when studying combination therapies. Our observation in the CAM tumor model is in agreement with most xenograft mouse models which show that the combination therapy has beneficial effects on tumor growth reduction 40–42. However, in the mouse studies, mostly only a concomitant treatment schedule was used. We now show that IR applied before sunitinib treatment can lead to better tumor growth reduction as compared to concomitant treatment. This is in agreement with two previous preclinical studies in mice 13,15 as well as with a clinical trial where it was suggested that sunitinib after radiotherapy is the main factor contributing to tumor response rates 43. All this further confirms the applicability of the CAM tumor model and the clinical relevance of addressing the scheduling of combination therapy.

The precise mechanism by which adjuvant sunitinib improves outcome compared to neo-adjuvant or concomitant treatment is subject for further investigations. Our data show that sunitinib decreases tumor cell proliferation and tumor vascularization. The latter was also observed in a breast cancer xenograft model and in patient tumor samples 44,45. The reduced vascularization could lead to increased hypoxia, as described in a melanoma xenograft model 46. Consequently, neo-adjuvant sunitinib might reduce therapeutic efficacy of IR due to increased hypoxia and reduced cell proliferation. In addition, IR might sensitize tumor cells to sunitinib or lead to an enhanced angiogenic response which is then counteracted by sunitinib. On the other hand, it has also been suggested that sunitinib could improve efficacy of IR by transiently inducing vessel normalization leading to better oxygenation of the tumor tissue 47,48. While we did not evaluate vessel normalization or tumor oxygenation in this study, our current results do not suggest that this mechanism occurred in this particular tumor model. Further research is required to unravel the exact underlying mechanism and to establish whether the current observations are a commonality when combining IR with angiostatic therapies.

An important finding of this study is that optimal scheduling of IR and sunitinib allows dose reduction without affecting the therapeutic benefit. To our knowledge, we are the first reporting on the effects of reducing the dose of sunitinib in combination with IR. This dose reduction is relevant for the clinical setting as there is a concern of increased side effects when both treatment modalities are combined, in particular with regard to bowel perforations and hemorragic events 17,43,49. The results of our study suggest that dose reduction in sunitinib in patients will not affect tumor response, whereas it might result in a better toxicity profile, provided that optimal scheduling is applied. In addition, when high dose radiotherapy is not possible for a patient, due to the risk of normal tissue toxicities, sunitinib could be added to the radiotherapy. This might allow reduction in the IR dose, without compromising the antitumor effect. To elucidate whether these dose reductions in the combination treatment have similar benefits in patients as we observed in our research, clinical trails are warranted.

Taken together, our results demonstrate that the CAM assay provides a feasible model to study the combination of different treatment modalities on angiogenesis and tumor growth, resulting in similar results as observed in other in vivo xenograft models. The major clinically relevant findings of our study are that precise scheduling of sunitinib and radiotherapy improves the therapeutic outcome and that this allows dose reduction in sunitinib without hampering the therapeutic efficacy. Further research should focus on the extension of different schedules and different doses of sunitinib and IR in order to improve the antitumor outcome with minimal toxicity. Especially, as our current results suggest that the maximum effective dose of sunitinib in combination with IR is significantly lower than the maximum tolerated dose. Furthermore, the most optimal and clinically relevant schedule should be tested in a clinical trial, with a focus on dosing and scheduling of both sunitinib and radiotherapy. This will lead to a better, faster, and more rational translation of this promising combination therapy to the clinic.

## Conflict of Interest

None declared.

## Supporting Information

**Figure S1.**The chicken chorioallantoic membrane model. (A) Images of ex ovo CAM development from EDD3 to EDD10 showing unidirectional growth of the vasculature with the embryo growing in the center of the CAM (arrow). (B) Representative image of CAM microvasculature analysis showing the different parameters that are acquired by the HetCAM software. (C) Vascular parameters obtained using HetCAM software during CAM development.

**Figure S2.**Dose–response analysis of sunitinib. (A) Vascular parameters obtained using HetCAM software on EDD10 following 4 days of treatment with sunitinib at the indicated dose. (B) Vascular parameters obtained using HetCAM software on EDD10 of the macrovasculature following 4 days of 50 *µ*L of 5.3 *µ*g/mL sunitinib. (C) Vascular parameters obtained using HetCAM software on EDD10 of the microvasculature following 2 days of 5.3 *µ*g/mL sunitinib.

**Figure S3.**Effect of irradiation on OE19 tumor growth in vivo. Tumor growth curves showing growth inhibition following single-dose irradiation (4 Gy) on EDD10. The bar graph shows the average weight of resected tumors (average + SD, *n* ≥ 6)

**Figure S4.**Proliferation, cell cycle, and apoptosis analysis for HT29 in vitro. (A) Proliferation assay, measuring luminescence of HT29 cells with CellTiterGlo. Cells either received 4 Gy or no irradiation. **P* < 0.05 (B) Cell cycle analysis of HT29 cells in vitro following 4 Gy IR, using PI FACS. (C) Apoptosis quantification of HT29 cells in vitro following 4 Gy IR, using PI FACS (D) Cell cycle analysis of HT29 cells in vitro following sunitinib treatment (1 *µ*mol/L), using PI FACS. **P* < 0.05

**Figure S5.**Effect of sunitinib on OE19 tumor growth in vivo. Tumor growth curves showing growth inhibition following treatment with sunitinib (20 *μ*g/day in 50 *μ*L) applied from EDD10-13. The bar graph shows the average weight of resected tumors (average + SD, *n* ≥ 6)

**Figure S6.**Tumor growth curves of HT29 following 2 Gy IR alone or in combination with sunitinib. (A) Tumor growth curves of HT29 showing growth inhibition following single-dose IR (2 Gy) on EDD10, 12 or 14. (B) Tumor growth curves of HT29 showing difference in growth inhibition following 2 Gy or 4 Gy single-dose IR on EDD10. (C) Tumor growth curves of HT29 showing growth inhibition following 4 Gy IR (EDD10) or 2 Gy IR (EDD10) in combination with sunitinib (10 or 20 *µ*g/day in 50 *µ*L EDD10-13).

**Figure S7.**Combination of radiation and sunitinib reduces the growth rate of OE19 tumor on the CAM more than either treatment alone. (A) Growth curves of OE19 tumors on CAM subjected to different treatment regimes. Treatment with sunitinib (10 *µ*g/day in 50 *µ*L) was applied from EDD10-13. Irradiation (2 Gy) was applied on EDD10. (B) Weight of OE19 tumors after resection on EDD17.
