# Supplementary figures and images for: Optimal treatment scheduling of ionizing radiation and sunitinib improves the antitumor activity and allows dose reduction
Source: Cancer Med. 2015 Mar 31;4(7):1003–15. doi: 10.1002/cam4.441 (PMC4529339; doi:10.1002/cam4.441)

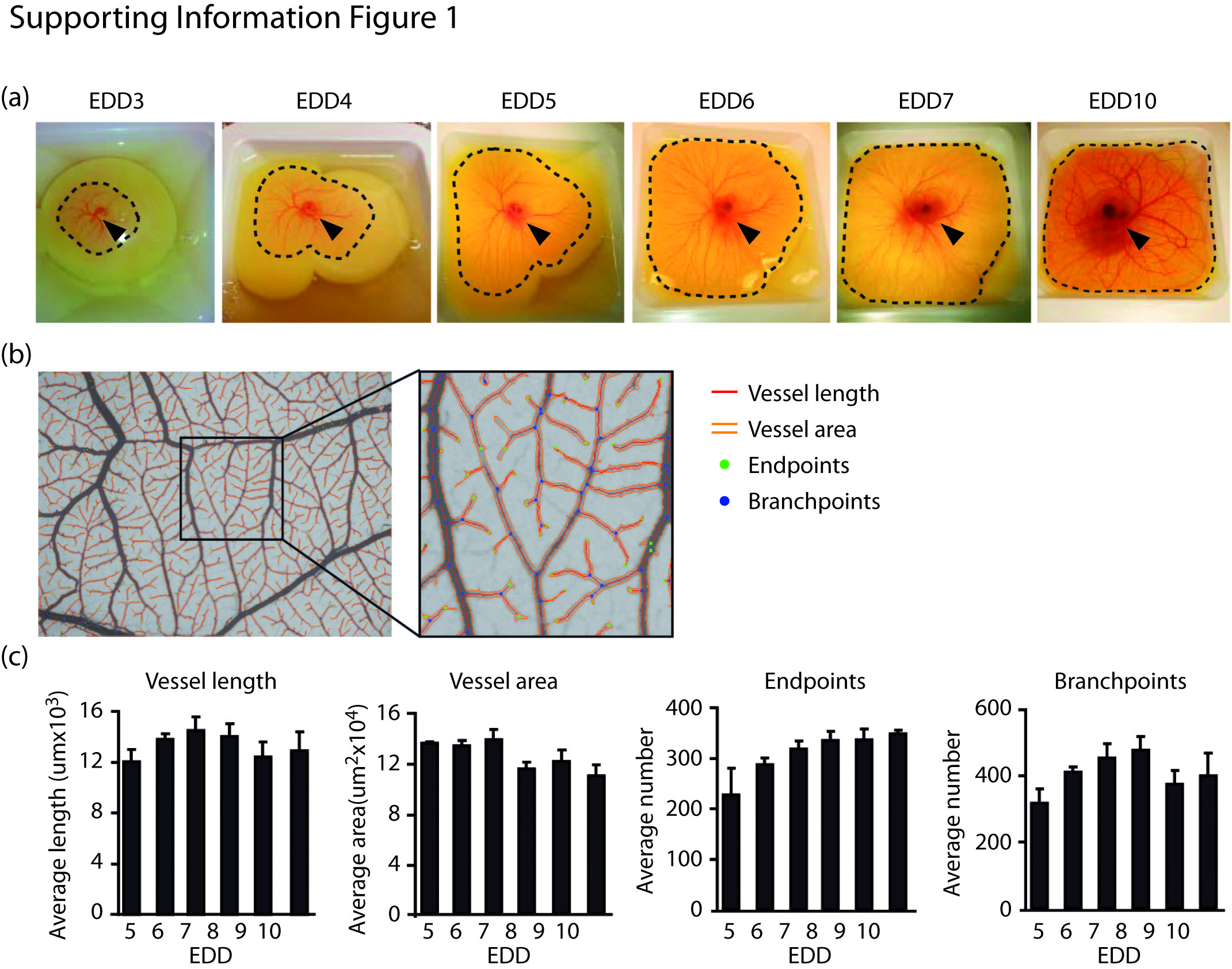

Supplement: Supplementary file 1 [file cam40004-1003-sd1.jpg]

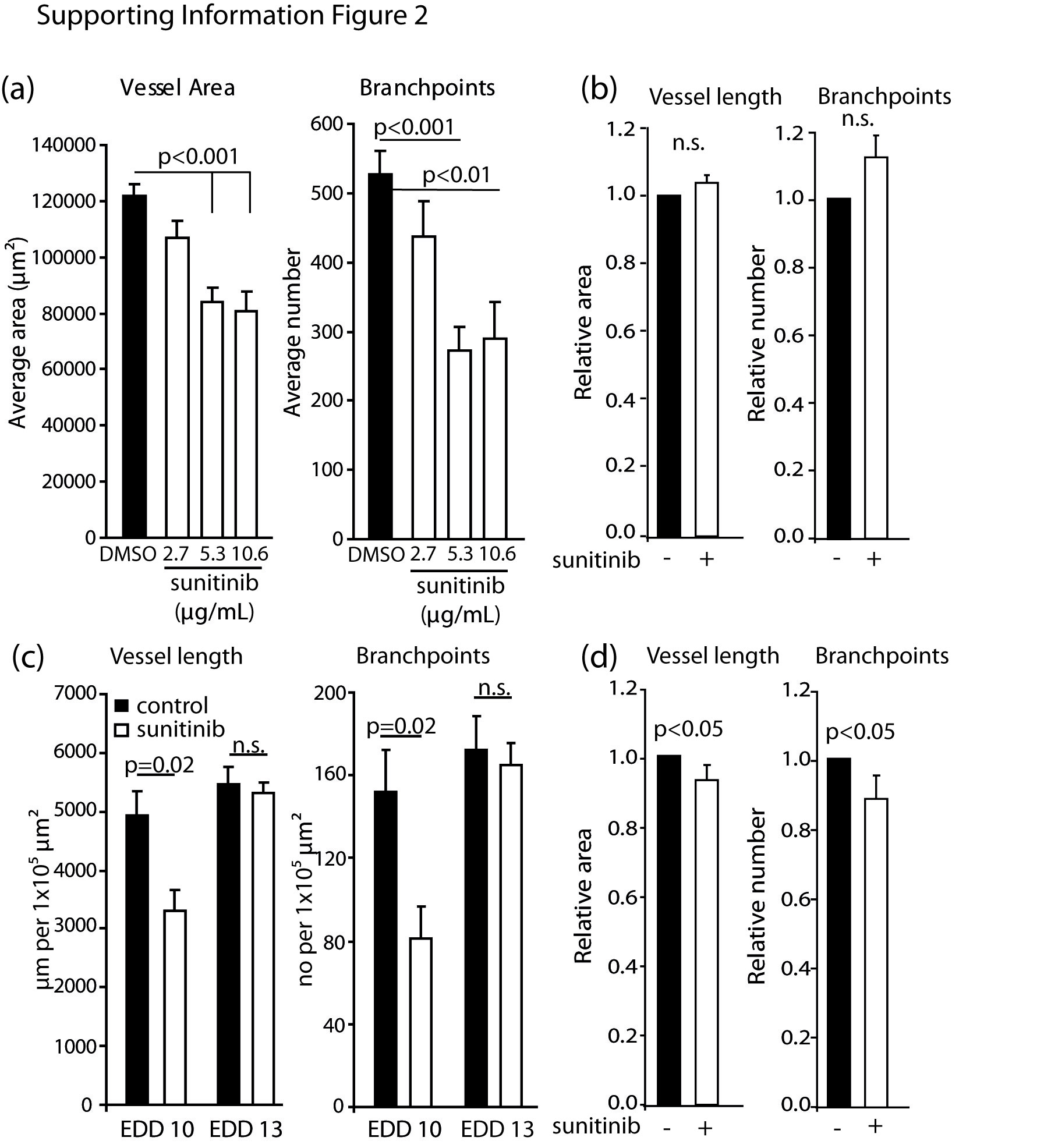

Supplement: Supplementary file 2 [file cam40004-1003-sd2.jpg]

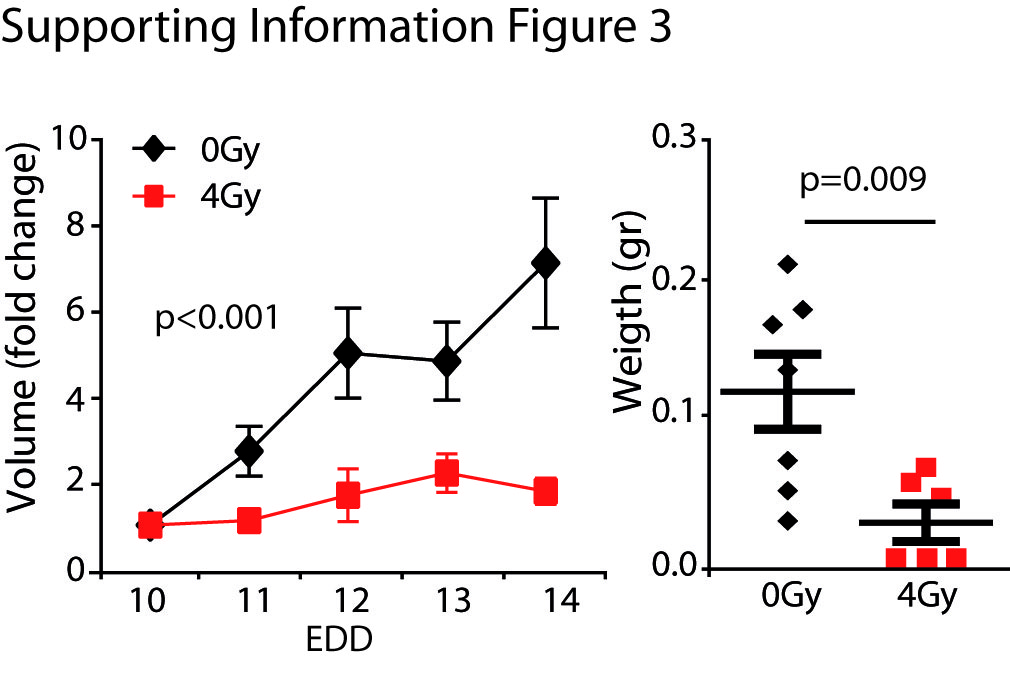

Supplement: Supplementary file 3 [file cam40004-1003-sd3.jpg]

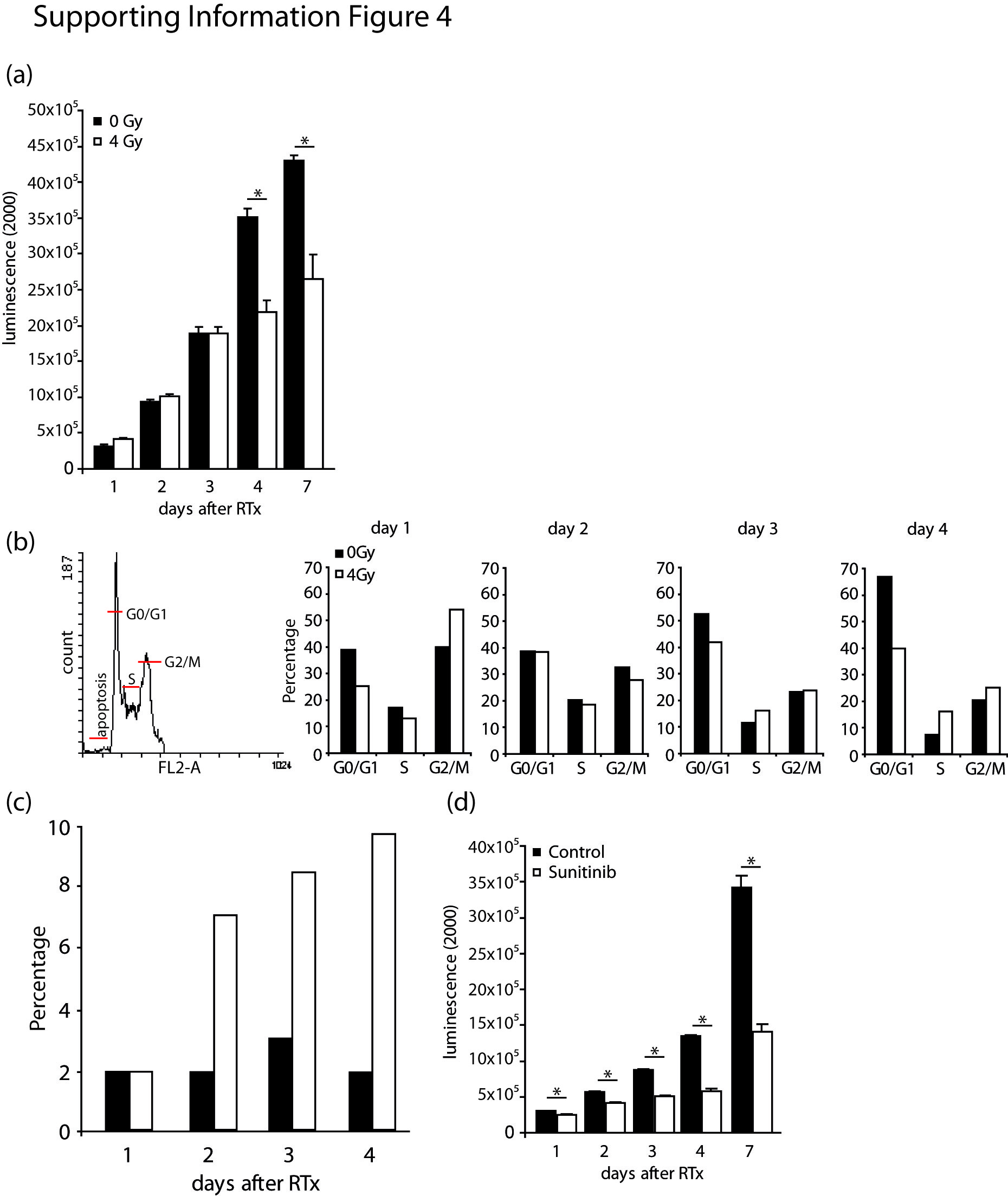

Supplement: Supplementary file 4 [file cam40004-1003-sd4.jpg]

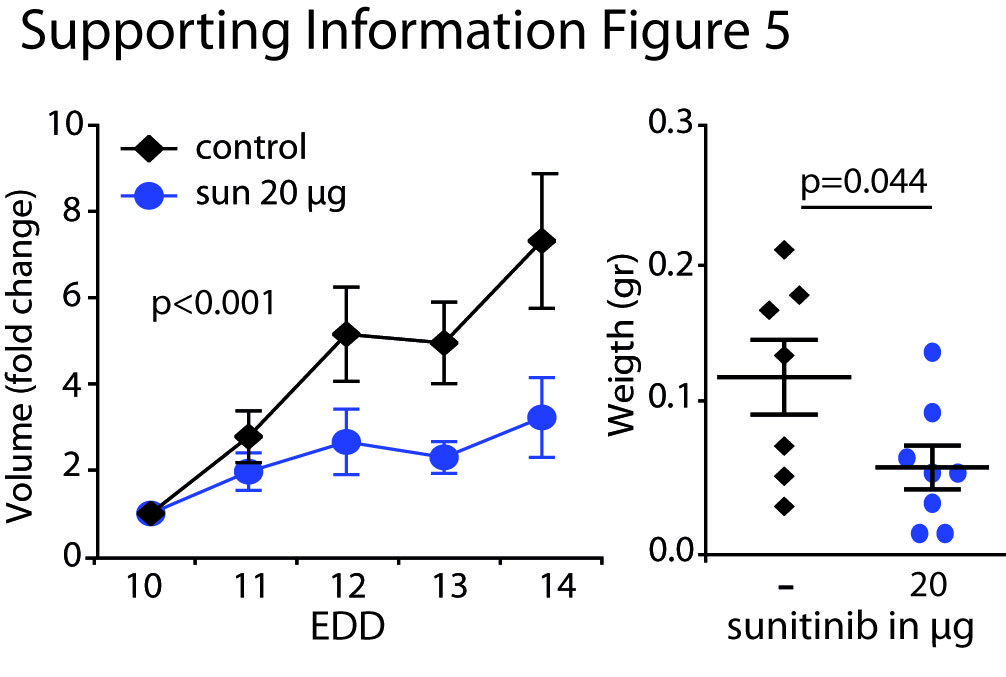

Supplement: Supplementary file 5 [file cam40004-1003-sd5.jpg]

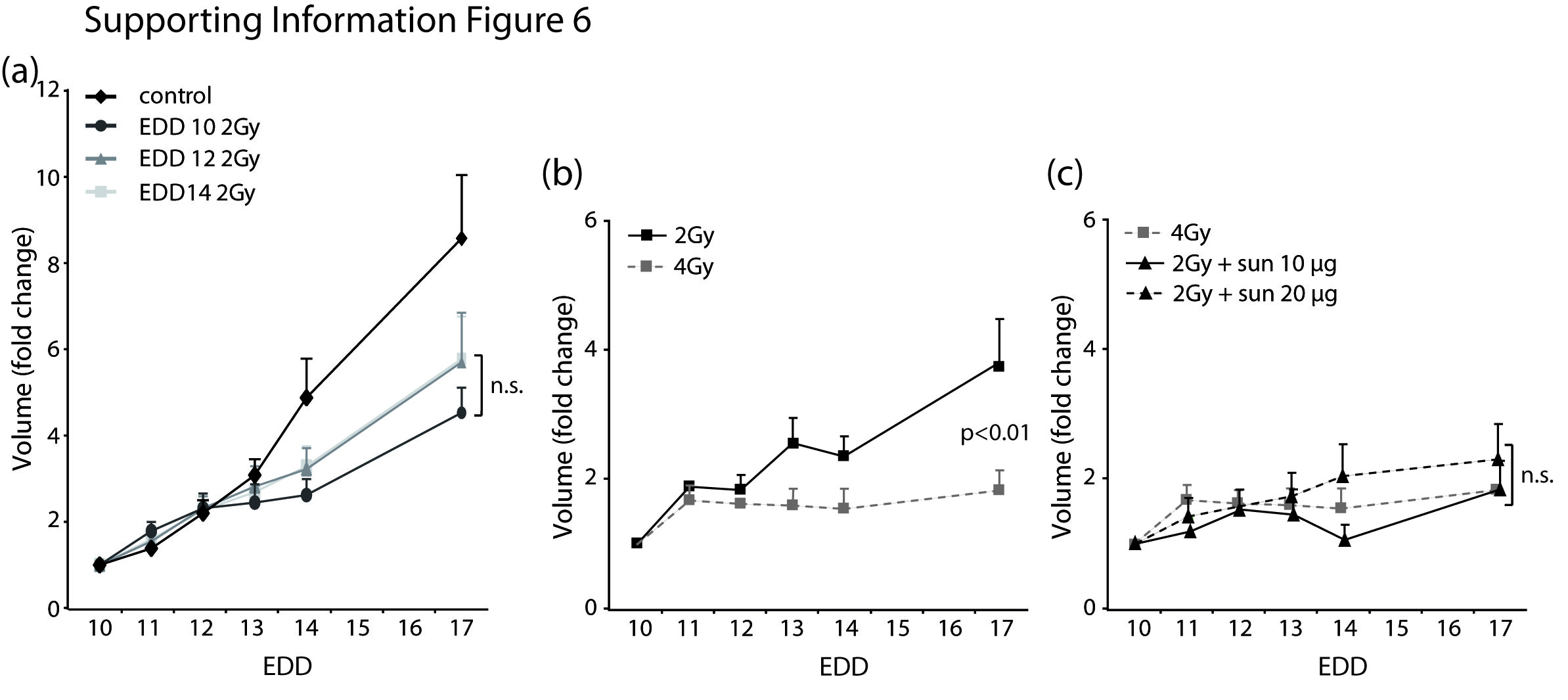

Supplement: Supplementary file 6 [file cam40004-1003-sd6.jpg]

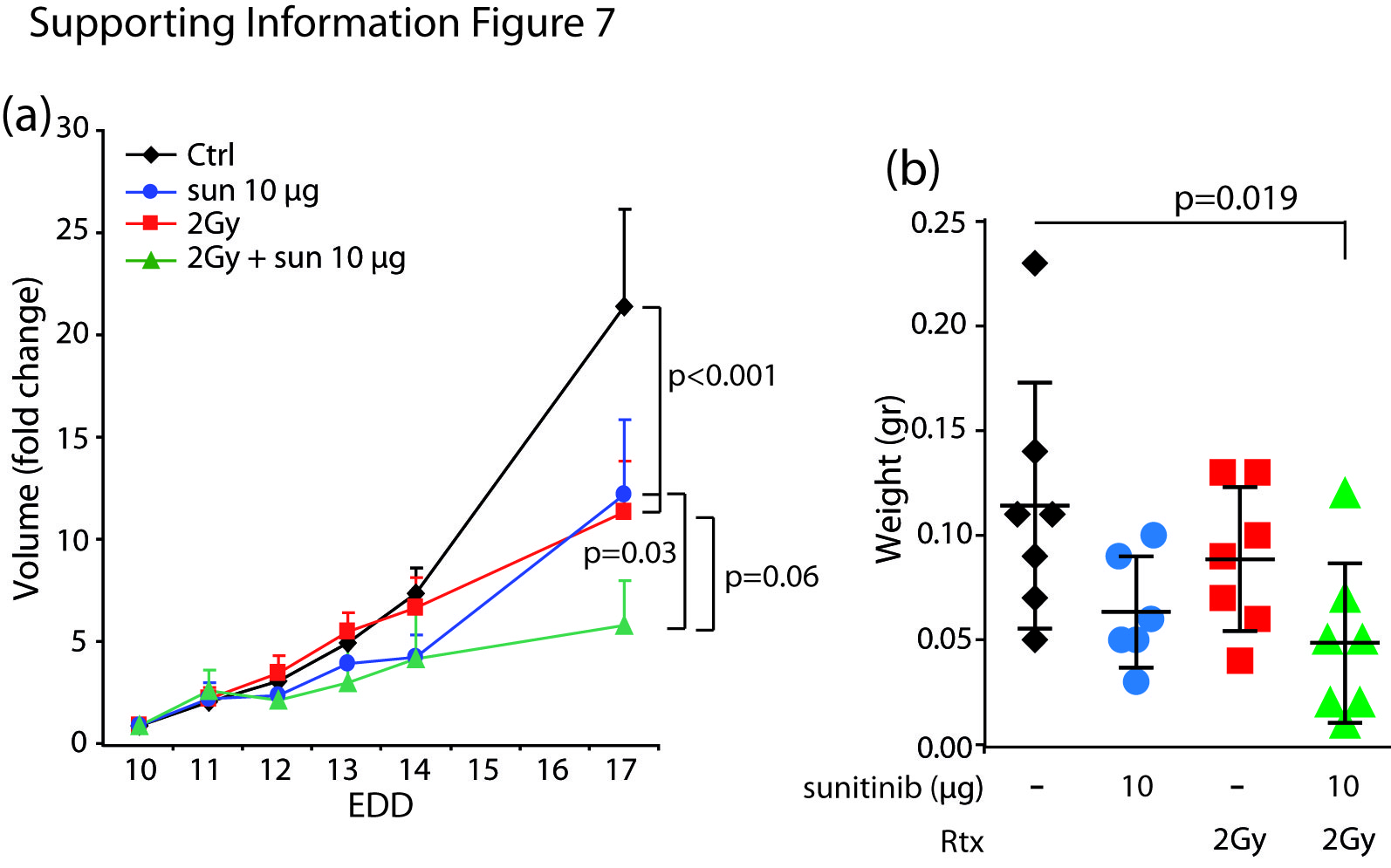

Supplement: Supplementary file 7 [file cam40004-1003-sd7.jpg]
