# Supplementary material for: Optimal treatment scheduling of ionizing radiation and sunitinib improves the antitumor activity and allows dose reduction
Source: Cancer Med. 2015 Mar 31;4(7):1003–15. doi: 10.1002/cam4.441 (PMC4529339; doi:10.1002/cam4.441)

**Supporting Information Figure 1.**

**The chicken chorioallantoic membrane model.**

(a) Images of ex ovo CAM development from EDD3 to EDD10 showing unidirectional growth of the vasculature with the embryo growing in the center of the CAM (arrow).

(b) Representative image of CAM microvasculature analysis showing the different parameters that are acquired by the HetCAM software.

(c) Vascular parameters obtained using HetCAM software during CAM development.

**Supporting Information Figure 2.**

**Dose-response analysis of sunitinib**

(a) Vascular parameters obtained using HetCAM software on EDD10 following 4 days of treatment with sunitinib at the indicated dose.

(b) Vascular parameters obtained using HetCAM software on EDD10 of the macrovasculature following 4 days of 50 µl of 5.3 µg/mL sunitinib.

(c) Vascular parameters obtained using HetCAM software on EDD10 of the microvasculature following 2 days of 5.3 µg/mL sunitinib.

**Supporting Information Figure 3.**

**Effect of irradiation on OE19 tumor growth in vivo.**

Tumor growth curves showing growth inhibition following single dose irradiation (4 Gy) on EDD10. The bar graph shows the average weight of resected tumors (average + SD, n ≥ 6)

**Supporting Information Figure 4.**

**Proliferation, cell cycle and apoptosis analysis for HT29 in vitro.**

(a) Proliferation assay, measuring luminescence of HT29 cells with CellTiterGlo. Cells either received 4 Gy or no irradiation. * p < 0.05

(b) Cell cycle analysis of HT29 cells in vitro following 4 Gy IR, using PI FACS.

(c) Apoptosis quantification of HT29 cells in vitro following 4 Gy IR, using PI FACS

(d) Cell cycle analysis of HT29 cells in vitro following sunitinib treatment (1 µM), using PI FACS. * p < 0.05

**Supporting Information Figure 5.**

**Effect of sunitinib on OE19 tumor growth in vivo.**

Tumor growth curves showing growth inhibition following treatment with sunitinib (20 ug/day in 50uL) applied from EDD10-13. The bar graph shows the average weight of resected tumors (average + SD, n ≥ 6)

**Supporting Information Figure 6.**

**Tumor growth curves of HT29 following 2 Gy IR alone or in combination with sunitinib.**

(a) Tumor growth curves of HT29 showing growth inhibition following single dose IR (2 Gy) on EDD10, 12 or 14.

(b) Tumor growth curves of HT29 showing difference in growth inhibition following 2 Gy or 4 Gy single dose IR on EDD10.

(c) Tumor growth curves of HT29 showing growth inhibition following 4 Gy IR (EDD10) or 2 Gy IR (EDD10) in combination with sunitinib (10 or 20 µg/ day in 50 µL EDD10-13).

**Supporting Information Figure 7**

**Combination of radiation and sunitinib reduces the growth rate of OE19 tumor on the CAM more than either treatment alone.**

(a) Growth curves of OE19 tumors on CAM subjected to different treatment regimes. Treatment with sunitinib (10 µg/ day in 50 µL) was applied from EDD10-13. Irradiation (2 Gy) was applied on EDD10.

(b) Weight of OE19 tumors after resection on EDD17.


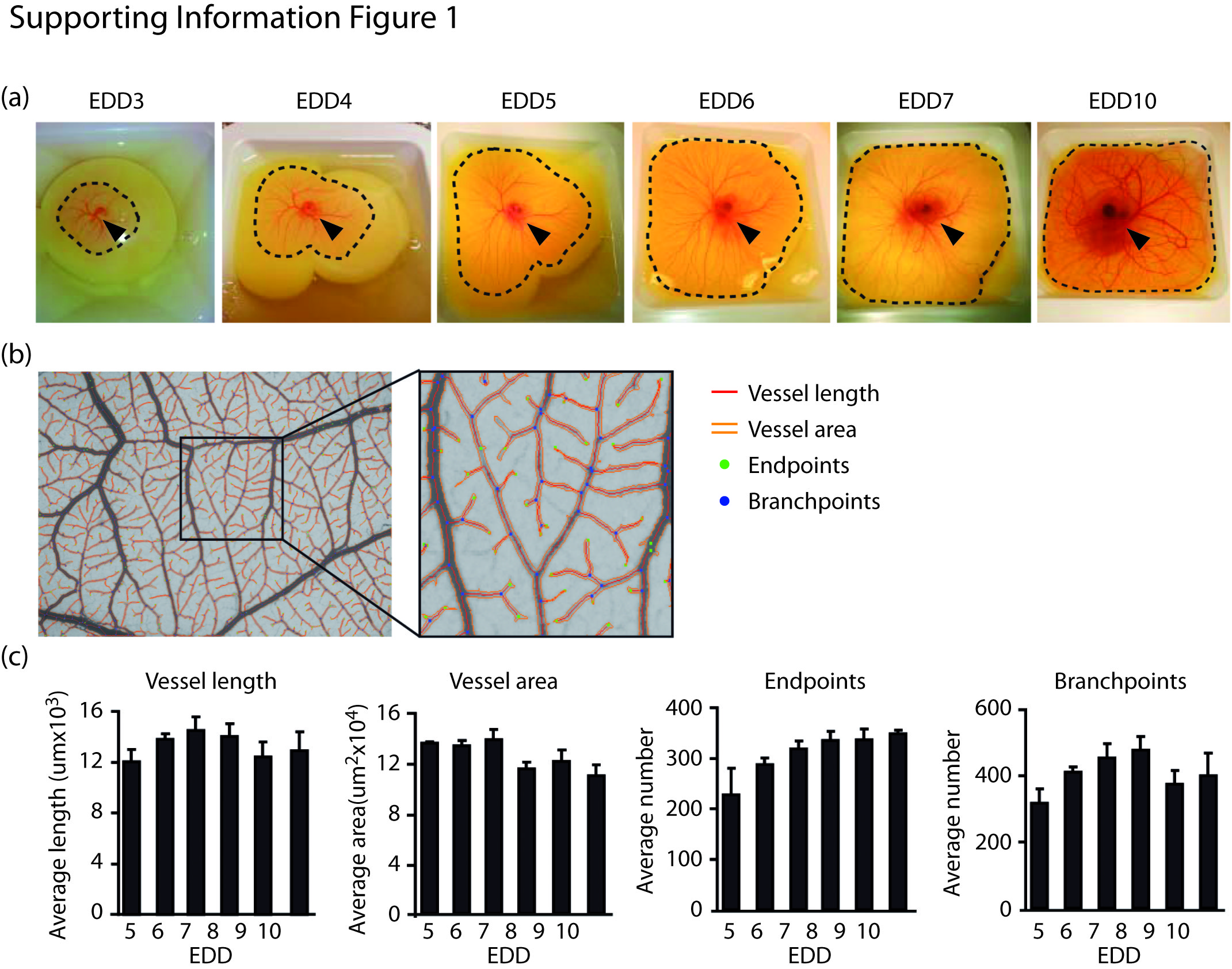


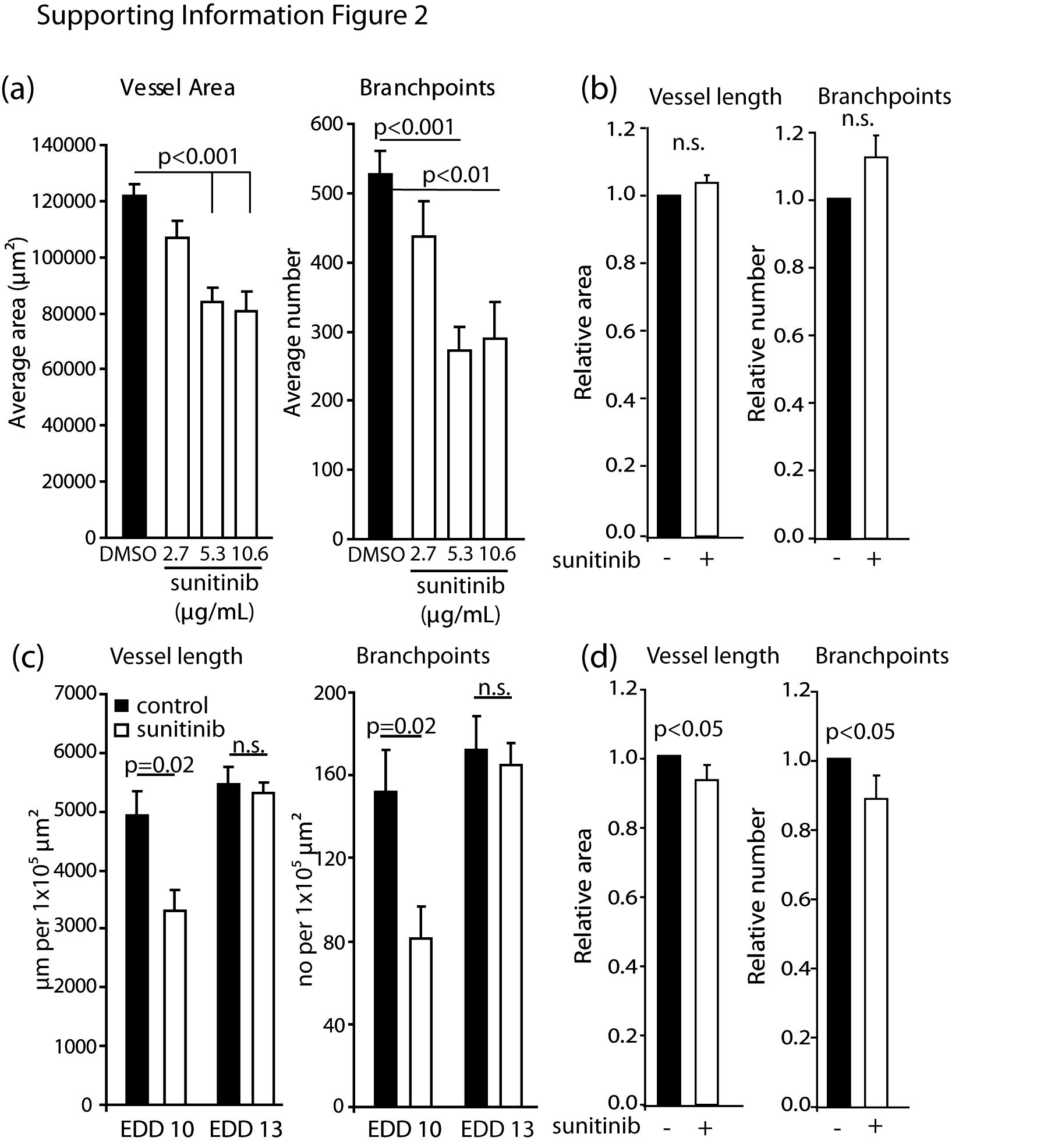


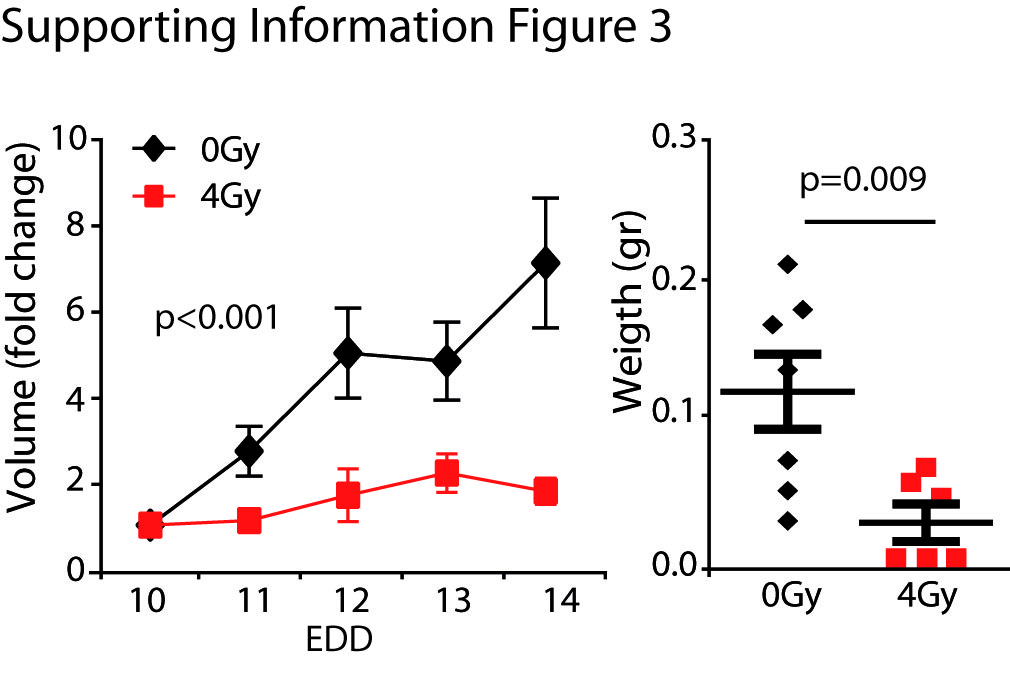


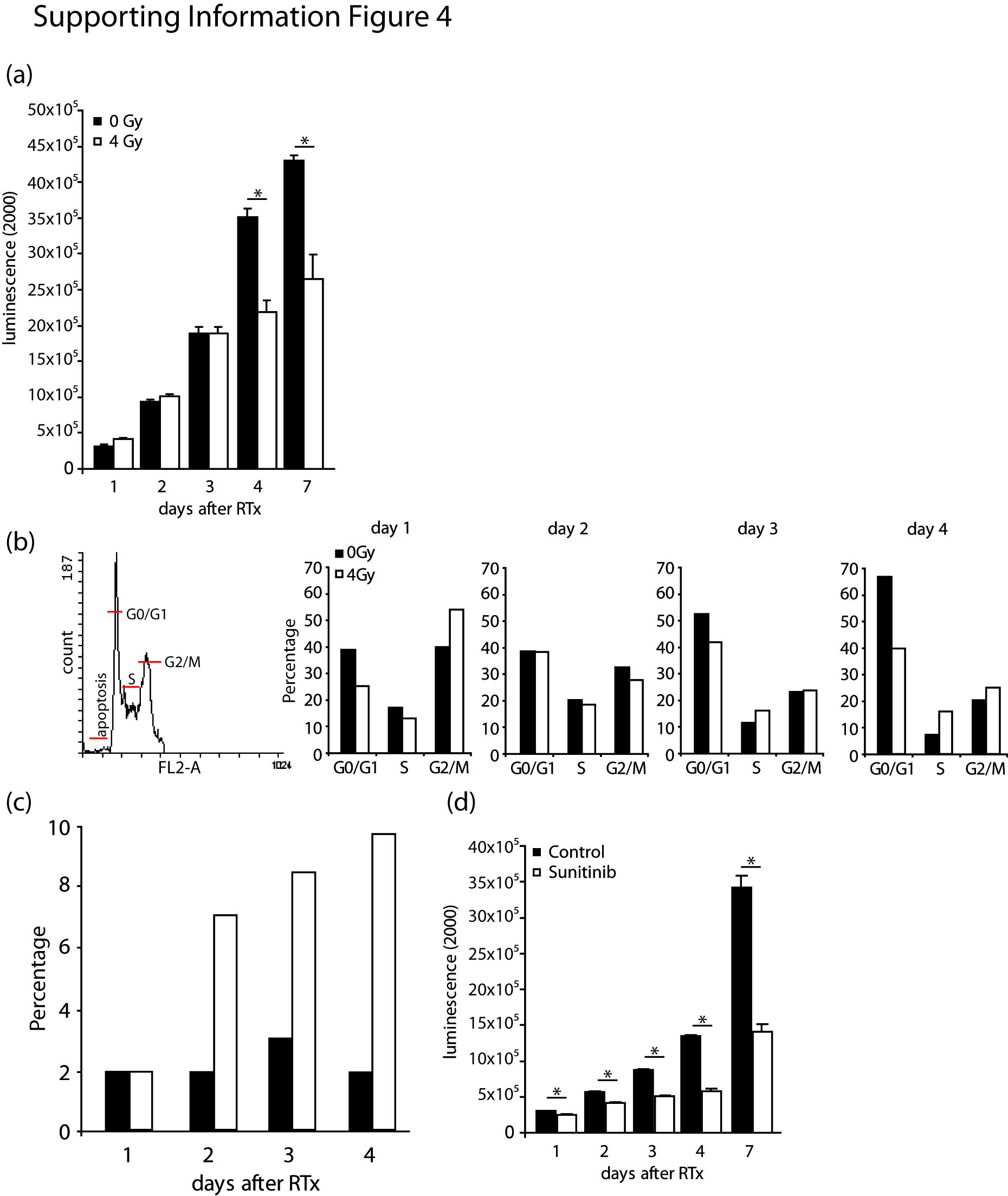


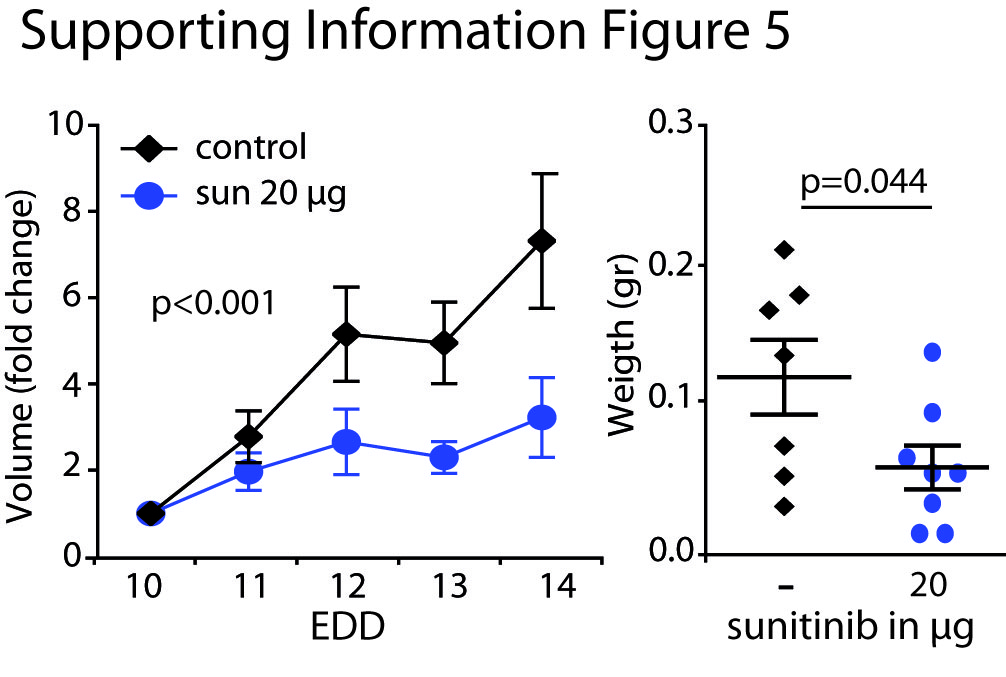


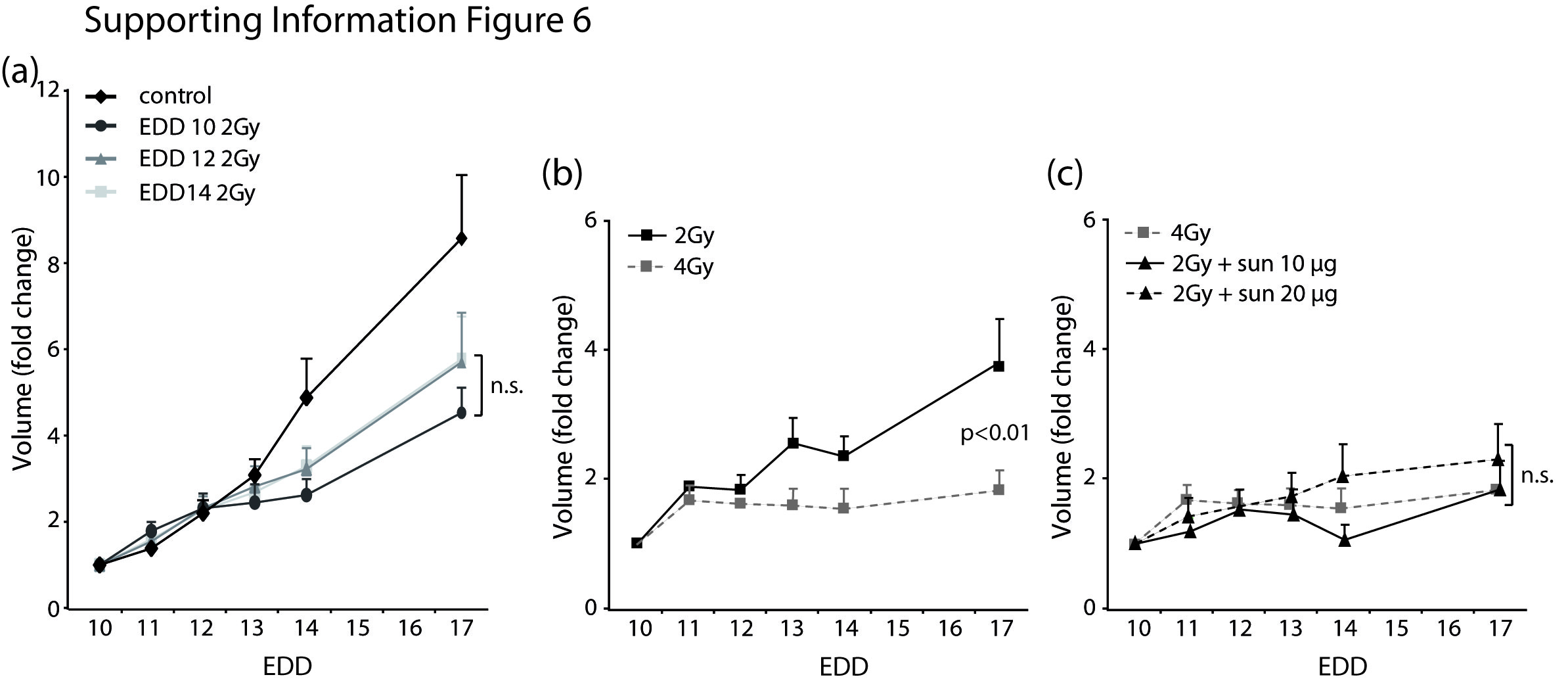

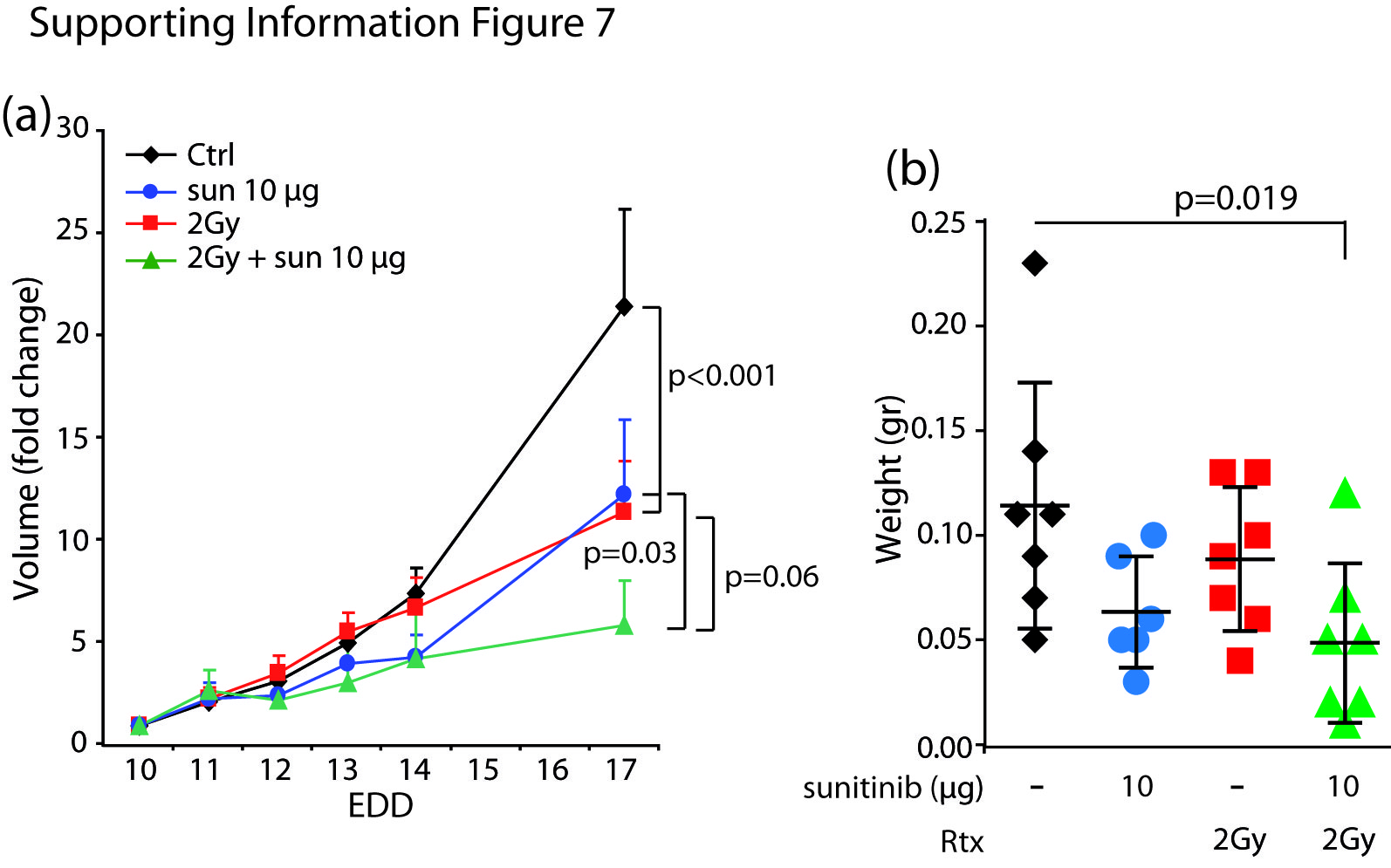

Supplement: Supplementary file 8 [file cam40004-1003-sd8.docx]
